# Electron-Beam-Driven Ag Nanoparticle Evolution at ZIF-8/Ag_3_PO_4_ Heterointerfaces

**DOI:** 10.1021/acs.langmuir.6c04357

**Published:** 2026-09-24

**Authors:** Júlia T. Ichikura, Gustavo H. F. Candido, Murilo Nader, Giovanna A. Grasser, Márcio D. Teodoro, Elson Longo, Henrique Moreno, Miguel A. San-Miguel

**Affiliations:** † CDMF-LIEC, Federal University of São Carlos (UFSCar), São Carlos 13565-905, Brazil; ‡ Institute of Chemistry, Universidade Estadual de Campinas (UNICAMP), Campinas 13083-970, Brazil; § Department of Physics,Federal University of São Carlos, São Carlos, SP 13565-905, Brazil

## Abstract

Silver orthophosphate (Ag_3_PO_4_) is a promising visible-light photocatalyst in which lattice Ag^+^ ions can be reduced to metallic Ag^0^ under external stimuli, but the subsequent evolution of these Ag species at heterogeneous interfaces remains poorly understood. Coupling Ag_3_PO_4_ with porous, electron-transport-active frameworks such as zeolitic imidazolate framework-8 (ZIF-8) offers a route to modulate this behavior, yet how reduced Ag species migrate across such heterointerfaces remains unexplored. Here, electron-beam irradiation (EBI) combined with time-resolved high-resolution transmission electron microscopy (HRTEM) reveals a previously unreported sequence of structural transformations in ZIF-8/Ag_3_PO_4_ heterostructures. Under continuous irradiation, reduced Ag species generated within Ag_3_PO_4_ diffuse through the ZIF-8 framework toward its outer surface, where they nucleate and grow into metallic Ag nanoparticles; prolonged exposure then drives their redistribution into the Ag_3_PO_4_ matrix, showing that this interfacial silver transport is fully dynamic. Density functional theory (DFT) calculations and ab initio molecular dynamics (AIMD) simulations reproduce the early stages of this process, revealing preferential electron accumulation within Ag_3_PO_4_, enhanced mobility of reduced Ag species, and the formation of Ag–Ag dimers as nucleation precursors. Together, these experimental and theoretical results establish a mechanism for electron-induced interfacial mass transport in MOF/semiconductor heterostructures, in which ZIF-8 acts not as a passive support but as an active medium that mediates silver migration and reversible interfacial reconstruction.

## Graphical abstract

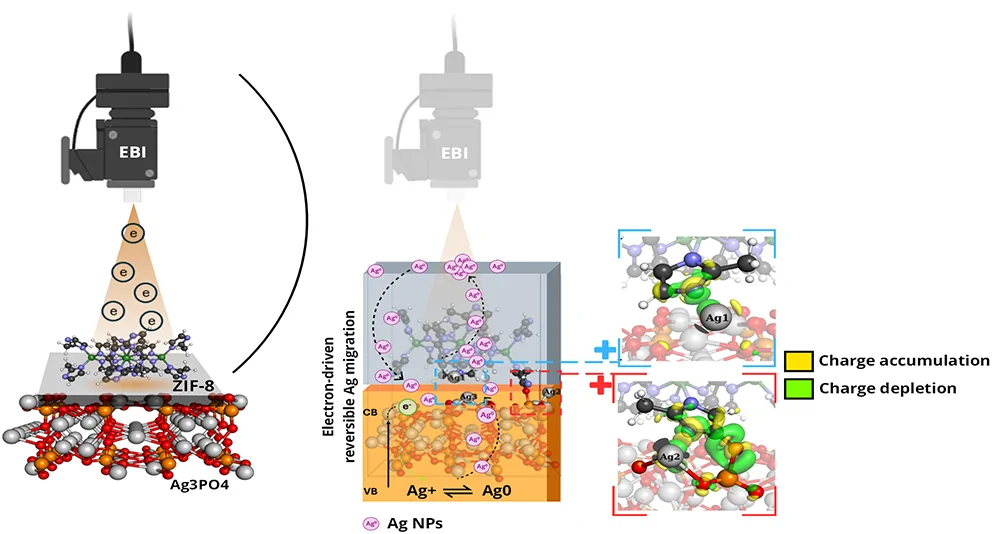

## Introduction

Metal–organic frameworks (MOFs) have emerged as one of the most versatile classes of porous crystalline materials owing to their tunable composition, high surface area, structural diversity, and tailorable physicochemical properties. These characteristics have enabled their widespread application in catalysis, gas adsorption and separation, sensing, energy storage, drug delivery, and photocatalysis.^1–4^ Among MOF subclasses, zeolitic imidazolate frameworks (ZIFs) have attracted particular attention for their exceptional thermal and chemical stability and well-defined porous architectures.^5,6^ In this context, ZIF-8, composed of Zn^2+^ nodes coordinated to 2-methylimidazolate ligands, stands out as one of the most extensively investigated ZIF materials.^7^ ZIF-8 exhibits a sodalite (SOD)-type topology, narrow microporous channels, and a high specific surface area (≈1408 m^2^ g^–1^), features that make it highly attractive for catalytic and interfacial engineering applications.^8^

Among Ag-based semiconductors, silver orthophosphate (Ag_3_PO_4_) has attracted considerable interest for its remarkable visible-light-driven photocatalytic activity, particularly in pollutant degradation and oxidation reactions.^9,10^ In addition, Ag_3_PO_4_ exhibits significant antimicrobial and bactericidal properties, broadening its potential technological applications.^11,12^ Structurally, Ag_3_PO_4_ is a p-type semiconductor with a body-centered cubic crystal structure and a relatively narrow band gap, with direct and indirect transition energies of approximately 2.36 and 2.43 eV, respectively.^13,14^ An important characteristic of Ag_3_PO_4_ is the susceptibility of lattice Ag^+^ species to reduction to metallic Ag^0^ under energetic excitation. Under photocatalytic conditions, this process is commonly associated with photocorrosion and can ultimately lead to structural degradation and semiconductor deactivation.^15,16^

Accordingly, considerable effort has focused on constructing Ag_3_PO_4_-based heterostructures and composites with other functional materials to modulate their interfacial, electronic, and functional properties.^17^ In particular, coupling Ag_3_PO_4_ with porous materials and electron-transport-promoting architectures has emerged as an effective strategy to modulate interfacial charge-transfer dynamics and stabilize reactive species.^18,19^ Although Ag^+^ reduction and subsequent formation of metallic Ag nanoparticles have been extensively reported in Ag_3_PO_4_-based systems, considerably less attention has been devoted to the spatial evolution of these species once generated, particularly their nucleation, redistribution, and growth across interfaces with initially Ag-free materials. Understanding such interfacial Ag dynamics is important because irradiation-induced redistribution of metallic Ag may locally restructure the heterointerface and consequently modify the physicochemical and multifunctional properties of the resulting heterostructure.

In this scenario, electron beam irradiation (EBI) has long been used in transmission electron microscopy (TEM) and scanning electron microscopy (SEM). Based on the same principle, EBI stands out as a versatile tool for the *in situ* synthesis, modification, and nanoengineering of materials at the atomic scale,^20^ as a result of electron-matter interactions; thereby inducing phenomena such as the nucleation, growth, migration, and coalescence of nanoparticles (NPs), as well as structural transformations that can be monitored in real time in response to external stimuli, without the need for chemical reagents and under high vacuum at room temperature.^21,22^

Our research group demonstrated the spontaneous nucleation and growth of metallic silver nanoparticles (NPs) on the surface of Ag_3_PO_4_ crystals under electron-beam irradiation (EBI), revealing the remarkable ability of lattice Ag^+^ ions to undergo reduction in response to external electronic excitation.^15^ Complementary *ab initio* molecular dynamics simulations based on density functional theory (DFT) provided unprecedented atomistic insight into the early stages of electron-induced silver reduction, atomic migration, and cluster nucleation.^23^ These findings established Ag_3_PO_4_ as a dynamic reservoir of highly mobile silver species capable of undergoing charge-driven structural reorganization. More broadly, they suggested, as other studies have, that electron irradiation can induce controlled structural transformations in silver-based materials.^24–27^ Nevertheless, a fundamental question remains unanswered: how do these processes evolve in complex hybrid systems? In particular, it remains unknown whether reduced Ag species generated within silver-based semiconductors can migrate across heterogeneous interfaces, interact with porous metal–organic frameworks, and ultimately drive the formation of interfacial metallic nanostructures. Answering this question is essential to establish the atomistic principles governing charge-induced interfacial reconstruction and to enable the rational design of dynamic hybrid materials.

In this work, we demonstrate that prolonged electron irradiation induces a remarkable sequence of structural transformations in the ZIF-8/Ag_3_PO_4_ heterostructure, including the nucleation, growth, migration, and eventual redistribution of Ag NPs into the Ag_3_PO_4_ matrix. Time-resolved high-resolution transmission electron microscopy (HRTEM) reveals that reduced silver species generated within Ag_3_PO_4_ migrate through the ZIF-8 framework toward its external surface, where they dynamically nucleate and grow into metallic Ag NPs. Remarkably, electron irradiation also promotes the reverse process, whereby the NPs are progressively reincorporated into the semiconductor, demonstrating that silver transport is fully dynamic and reversible under electron-beam excitation. Complementary DFT calculations reproduce the initial stages of this process by revealing preferential electron accumulation within Ag_3_PO_4_, enhanced mobility of reduced Ag species, and the formation of Ag–Ag dimers that act as precursors to metallic silver nucleation. Together, the experimental and theoretical results uncover a previously unreported mechanism of electron-induced interfacial mass transport in MOF/semiconductor heterostructures, involving coupled charge transfer, silver reduction, atomic diffusion, and reversible structural reconstruction. Beyond elucidating the fundamental physics of this dynamic interface, this work establishes ZIF-8 not merely as a passive support but as an active medium that mediates silver transport and interfacial reconfiguration under electronic excitation.

## Materials and Methods

### Synthesis

Zeolitic imidazolate framework-8 (ZIF-8) NPs were prepared through a microwave-assisted solvothermal (MAS) approach employing zinc nitrate hexahydrate (Zn­(NO_3_)_2_·6H_2_O, Chem Impex Inc., 99.84%) and 2-methylimidazole (2-mIm, Sigma-Aldrich, 99.0%) as starting materials. Initially, the zinc precursor was dissolved in 20 mL of N,N-dimethylformamide (DMF, Neon, 99.8%) under continuous magnetic stirring at ambient conditions for 20 min to ensure complete solvation. In a separate vessel, 2-mIm was dissolved in 30 mL of DMF at 40 °C under constant stirring until a homogeneous solution formed. Subsequently, the ligand solution was gradually added to the zinc-containing solution under vigorous stirring, followed by an additional 30 min of stirring to facilitate coordination interactions and nucleation. The resulting reaction mixture was then transferred to a Teflon-lined autoclave and subjected to microwave-assisted heating at 120 °C for 32 min, using a 2 min heating ramp. After the reaction, the system was allowed to cool naturally to room temperature. The resulting powder was recovered, thoroughly washed with ethanol (three cycles) to remove residual species, and dried in an oven overnight to obtain the final ZIF-8 nanopowder.

Silver phosphate (Ag_3_PO_4_) particles were synthesized via a coprecipitation route using silver nitrate (AgNO_3_, Sigma-Aldrich, 99.0%) and diammonium hydrogen phosphate (NaH_2_PO_4_·H_2_O, Mallinckrodt, 98.8%) as precursors, following a protocol reported by Botelho et al.^9^ Initially, each precursor was independently dissolved in 50 mL of deionized water under constant magnetic stirring to ensure complete dissolution and uniform dispersion. The solutions were then quickly mixed, yielding a yellow precipitate. The resulting Ag_3_PO_4_ powder was centrifuged, washed 3 times with ultrapure water, and then dried in an oven at 60 °C overnight.

The ZIF-8/Ag_3_PO_4_ heterostructure was prepared by combining previously synthesized ZIF-8 and Ag_3_PO_4_ powders in a 1:1 weight ratio, as reported in previous works by our group.^28,29^ The resulting powder mixture was dispersed in water and ultrasonicated for 5 h in an ultrasonic bath to promote homogeneous dispersion and intimate contact between the ZIF-8 and Ag_3_PO_4_ particles. No uncommon hazards are noted.

### Characterization

We systematically investigated the structural features of all samples across short-, medium-, and long-range order using Fourier-transform infrared (FTIR), Raman, and photoluminescence (PL) spectroscopy as well as X-ray diffraction (XRD). FTIR measurements were conducted at room temperature employing a Jasco FT/IR-6200 spectrometer operating in attenuated total reflectance (ATR) mode. Spectra were acquired over the 400–4000 cm^–1^ range with a spectral resolution of 4 cm^–1^, averaging 64 scans per measurement to enhance the signal-to-noise ratio. Micro-Raman spectroscopy measurements were carried out using a LabRAM iHR550 spectrometer (Horiba Jobin-Yvon, Japan) equipped with a charge-coupled device (CCD) detector and a He–Ne laser (MellesGriot, USA) as the excitation source, operating at 633 nm with a power of 200 mW. The spectral data were collected over the range 50–1000 cm^–1^. PL spectroscopy was carried out using a 355 nm diode laser (Cobolt/Zouk) as the excitation source, with an incident power of 1 mW at the sample surface. Finally, X-ray diffraction patterns were recorded using a Rigaku SmartLab SE diffractometer, with data collected over a 2θ range of 5° to 80° at a scan rate of 5° min^–1^.

Evaluation of particle morphology was carried out using a combination of scanning electron microscopy (FEG-SEM) and transmission electron microscopy (TEM), performed in a Supra 35 microscope operating at 5 kV with a field-emission gun (FEG) and in a cold FEG Talos (F200X, FEI) TEM microscope. EBI dynamics were assessed using the FEG-SEM microscope, with sequential micrographs recorded at 1 min intervals over 5 min. These experiments highlighted Ag NP formation. Analogously, TEM micrographs were recorded at 200 kV (143 pA) over an area of 289 × 289 nm, yielding a 4.5 × 10^3^ e^–^. nm^2^ t electron dose.

### Computational Details

All calculations were performed using the Vienna Ab Initio Simulation Package (VASP) within the framework of density functional theory (DFT). The exchange–correlation energy was described using the generalized gradient approximation (GGA) with the Perdew–Burke–Ernzerhof (PBE) functional. Long-range dispersion interactions were accounted for using the Grimme D3 correction. Core and valence electron interactions were treated using the projector-augmented-wave (PAW) method. A plane-wave basis set with a kinetic energy cutoff of 450 eV was employed throughout all calculations.

Geometry optimizations were carried out using the conjugate-gradient algorithm until the residual forces on all atoms were smaller than 0.05 eV Å^–1^, while the electronic self-consistent field (SCF) convergence criterion was set to 1 × 10^–5^ eV. Owing to the large dimensions of the surface and heterostructure supercells, Brillouin-zone integrations were performed using a Γ-centered (1 × 1) Monkhorst–Pack grid. A vacuum region larger than 15 Å was introduced along the nonperiodic direction to avoid interactions between periodic images.

The Ag_3_PO_4_ (110) surface was selected based on our previous experimental and theoretical investigation of the response of low-index Ag_3_PO_4_ facets to electron-beam irradiation.^23^ In that study, we compared the (001), (110), and (111) surfaces, and the (110) facet showed the most pronounced structural transformations under electron irradiation. These transformations were associated with the higher density of undercoordinated surface Ag^+^ sites on the (110) facet, which provide preferential sites for electron-induced reduction and subsequent structural reorganization. This feature is particularly relevant to the present work, which focuses on the reduction, migration, and aggregation of Ag species under electron-rich conditions. In addition, previous studies have suggested that the Ag_3_PO_4_ (110) facet exhibits particularly high photocatalytic activity among the low-index surfaces.^13,14,30^ The combination of its high density of undercoordinated Ag^+^ sites, pronounced response to electron irradiation, and reported photocatalytic activity therefore motivated its selection as the Ag_3_PO_4_ surface model in the present study.

The Ag_3_PO_4_ surface model was generated by cleaving the fully optimized bulk structure along the (110) crystallographic plane. The ZIF-8 model was derived from its crystallographic structure. A finite cluster of 135 atoms was extracted from the bulk framework to preserve the local coordination environment of the Zn centers and the connectivity of the 2-methylimidazolate linkers. The resulting cluster was subsequently optimized prior to the construction of the Ag_3_PO_4_/MOF heterostructure. This cluster approach enables the description of the local interfacial interactions between the semiconductor surface and the ZIF-8 while maintaining a computationally tractable model. This approximation may introduce finite-size and surface-termination effects that can influence the absolute adsorption energies, local electrostatic potential, and distribution of electronic charge within the MOF. In particular, the atoms at the cluster boundary may possess electronic environments that differ from those in bulk ZIF-8, and therefore their contribution to the calculated charge redistribution should be interpreted with caution. Nevertheless, the structural and electronic effects discussed here are primarily associated with the interfacial region, whereas the outermost cluster atoms are spatially separated from the ZIF-8/Ag_3_PO_4_ contact. ZIF-8/Ag_3_PO_4_ heterostructures were obtained by placing the MOF cluster atop the Ag_3_PO_4_ (110) surface and then performing full structural optimization. The adsorption energy *E*_ads_ was calculated according to eq 1

1 $$E_{ads}=E_{heterostructure}-E_{{Ag}_{3}{PO}_{4}}-E_{ZIF‐8}$$

where *E*_heterostructure_, *E*_Ag3PO4_ and *E*_ZIF‑8_ correspond to the total energies of the optimized heterostructure, isolated Ag_3_PO_4_ slab, and isolated MOF, respectively

Electron irradiation effects were simulated by progressively increasing the total number of electrons in the system through the NELECT tag implemented in VASP. In periodic boundary conditions, the added charge is compensated by a uniform neutralizing background, allowing the investigation of increasingly electron-rich conditions. Ab initio molecular dynamics (AIMD) simulations were performed within the canonical (NVT) ensemble using a Nosé–Hoover thermostat and Γ-point sampling. The structural evolution of the irradiated heterostructures was monitored through atomic trajectories, mean-square displacements (MSD), and radial distribution functions (*g*(*r*)). Charge transfer processes were analyzed using the Bader partitioning scheme applied to the converged charge densities. The resulting atomic charges were employed to quantify charge redistribution between the Ag_3_PO_4_ surface and the MOF as a function of electron injection and structural evolution.

## Results and Discussion

The successful construction of the ZIF-8/Ag_3_PO_4_ heterostructure, while preserving the structural integrity of its constituent phases, is evidenced by the convergence of diffraction, vibrational, and microscopic characterization results, further substantiated by first-principles calculations. Figure 1A–C presents a comprehensive structural, vibrational, electronic, and morphological characterization of pristine ZIF-8, Ag_3_PO_4_, and the ZIF-8/Ag_3_PO_4_ heterostructure, showing successful integration of both phases and revealing interfacial electronic interactions established after heterojunction formation. The combined analysis strongly supports the existence of an electronically coupled hybrid system that promotes charge redistribution at the interface, with potential applications for modulating surface-based properties and the composite’s physicochemical behavior. The X-ray diffraction (XRD) patterns shown in Figure 1A confirm the preservation of the crystalline structures of both constituents after heterostructure assembly, showing peaks associated with ZIF-8 (ICSD n. 5028256) with sodalite-type topology, indicating that the MOF retains its long-range crystallographic order after hybridization. Simultaneously, the diffraction reflections corresponding to Ag_3_PO_4_ (ICSD n. 140001) – with *P*4̅3n space group in a cubic system—are clearly identified and remain well-defined in the composite sample, demonstrating that the phosphate phase also preserves its crystalline integrity. Importantly, no secondary phases or impurity-related reflections are observed, indicating that the synthesis route promotes direct coupling between ZIF-8 and Ag_3_PO_4_ without inducing structural decomposition or undesirable side reactions. The coexistence of both sets of diffraction peaks in the composite provides direct evidence for successful heterostructure formation.

**Figure 1:**
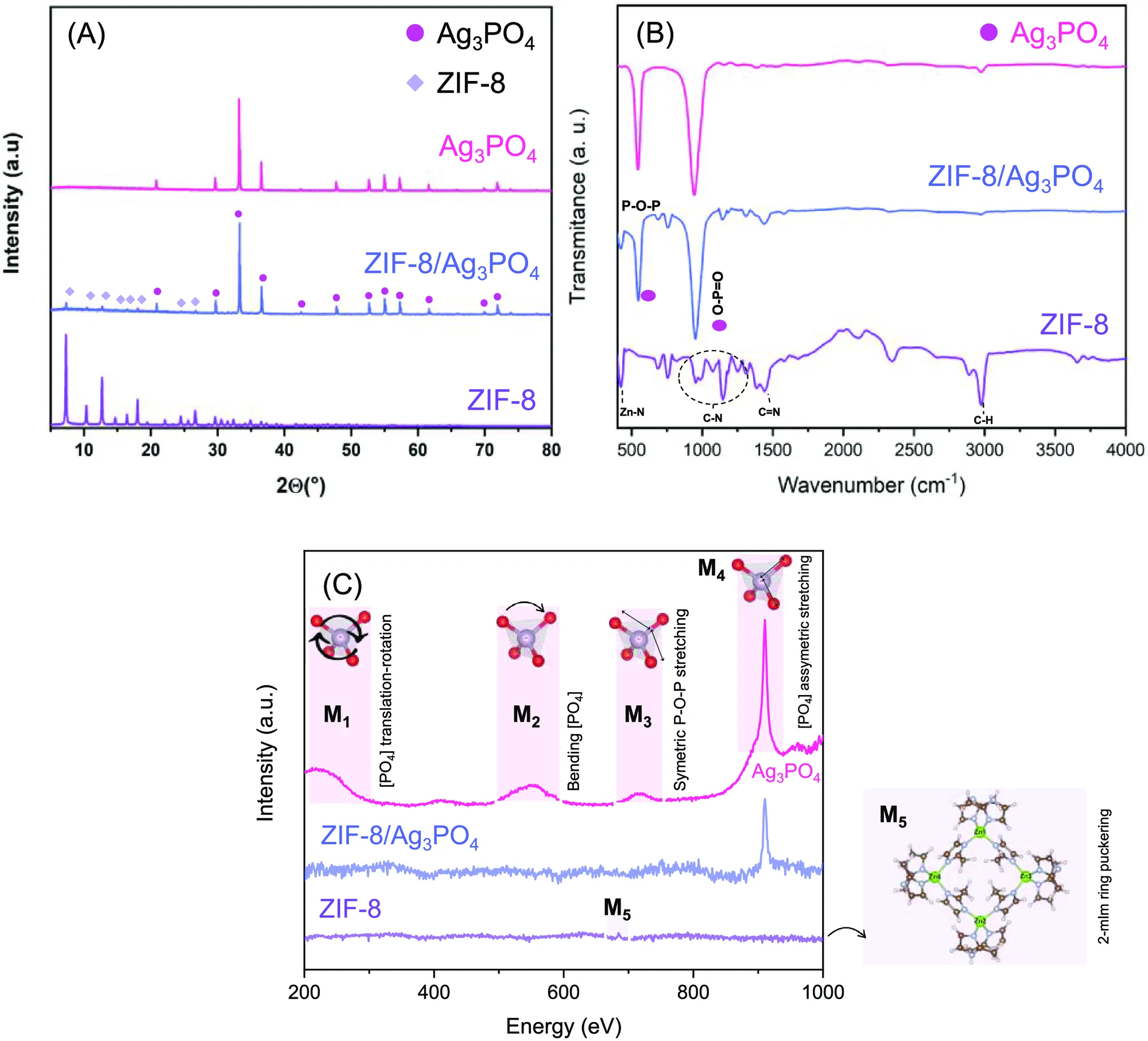
Comparison between the (A) XRD diffractograms, (B) FTIR, and (C) Raman, showing the evolution of the long-, medium-, and short-range order for the ZIF-8/Ag_3_PO_4_ heterostructure and its pristine constituents.

The Fourier-transform infrared (FTIR) spectra displayed in Figure 1B further corroborate the structural integration between ZIF-8 and Ag_3_PO_4_. The vibrational bands characteristic of ZIF-8, particularly those associated with the imidazolate linker vibrations, Zn–N coordination modes, and C–N stretching vibrations, remain preserved in the heterostructure, confirming that the framework connectivity is maintained after composite formation. In parallel, the absorption features attributed to phosphate-related vibrational modes from Ag_3_PO_4_ are also detected in the ZIF-8/Ag_3_PO_4_ sample. Notably, the heterostructure spectrum shows subtle band shifts and intensity variations relative to the pristine materials, suggesting interfacial electronic interactions between the MOF and semiconductor phases. These modifications are consistent with local perturbations in bond polarization and electron-density redistribution induced by heterointerface formation. Moreover, the Raman spectra in Figure 1C provide additional insight into short-range structural organization and interfacial coupling effects. The pristine Ag_3_PO_4_ spectrum exhibits the characteristic vibrational modes associated with the PO_4_^3–^ tetrahedral units. The comparatively weak Raman features of ZIF-8 should be interpreted in light of the pronounced background observed under 532 nm excitation, which is most likely associated with fluorescence and substantially reduces the Raman signal-to-background ratio. As a consequence, most of the characteristic ZIF-8 vibrational modes are poorly resolved under the present experimental conditions, although a weak contribution at approximately 680 cm^–1^ (M_5_) can still be identified. Specifically, at 909 cm^–1^ (M_4_), the vibrational mode is attributed to the symmetric stretching of [PO_4_^3–^] clusters. Analogously, the mode at 710 cm^–1^ (M_3_) can be ascribed to the symmetric stretching vibration P–O–P. The band at 406 cm^–1^ (M_2_) is attributed to the bending of [PO_4_^3–^] clusters, and the [PO_4_^3–^] rotation mode can be identified at 223 cm^–1^ (M_1_). Finally, a vibrational mode at approximately 685 cm^–1^ corresponds to the imidazolium ring puckering in ZIF-8 (M_5_).

The photoluminescence (PL) spectra shown in Figure 2A–C provide important insights into the degree of structural order/disorder and the presence of vacancy-related defects in the pristine materials and heterostructure.^31–33^ Ag_3_PO_4_ exhibits a dominant broad emission centered in the visible region, which is commonly associated with defect-mediated radiative recombination involving oxygen vacancies and distorted [AgO_4_]/[PO_4_] clusters. In contrast, ZIF-8 exhibits a distinct emission profile associated with ligand-centered electronic transitions and its comparatively higher local structural order. After heterostructure formation, the ZIF-8/Ag_3_PO_4_ exhibits a significant redistribution of the PL emission components, accompanied by notable changes in the relative integrated areas. This behavior indicates a change in the local electronic environment and defect population at the interface.

**Figure 2:**
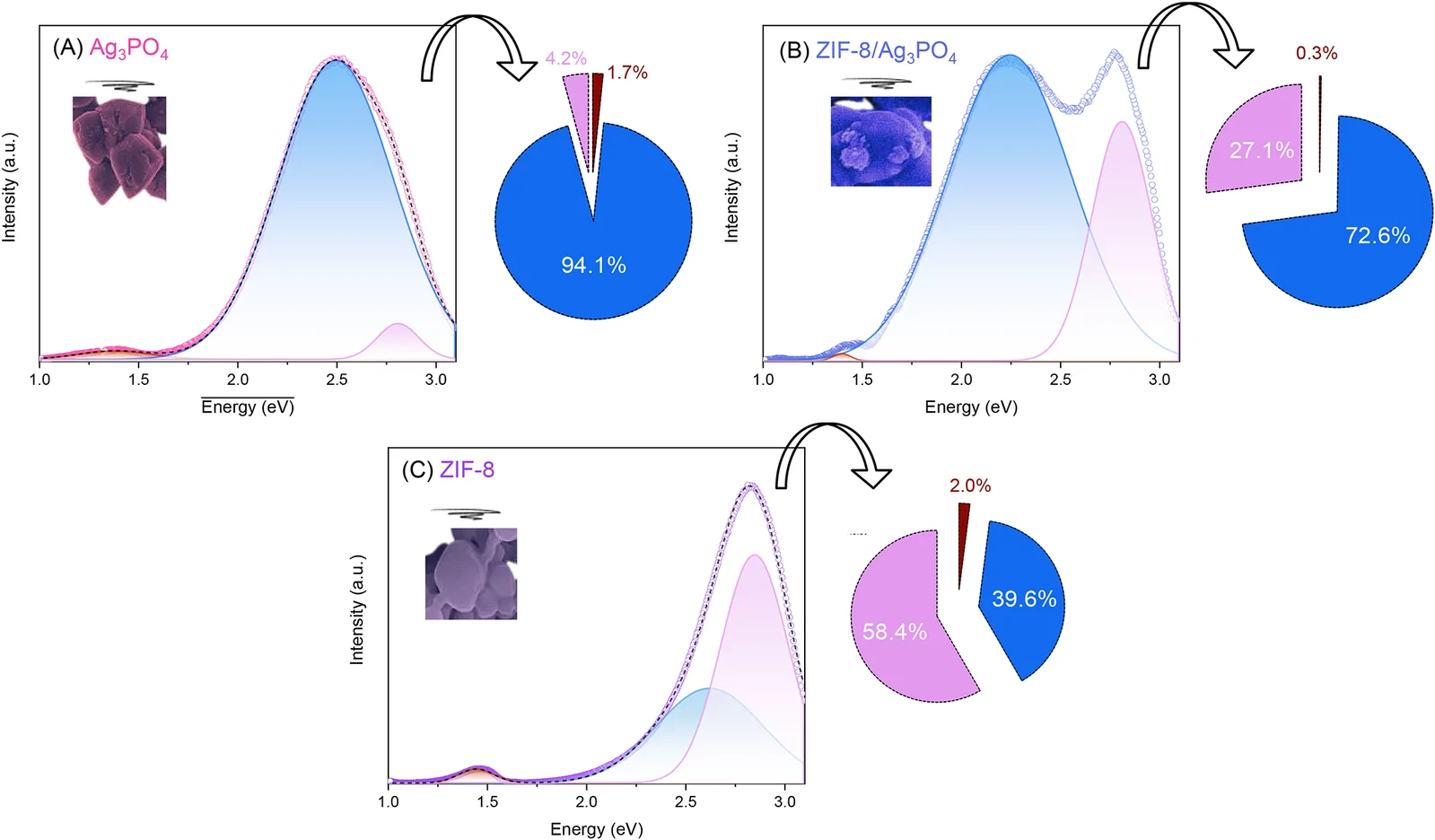
PL spectrum deconvolution for sample (A) Ag_3_PO_4_, (B) ZIF-8, and (C) ZIF-8/Ag_3_PO_4_.

These experimental observations are corroborated by density functional theory (DFT) calculations, which provide an atomistic rationale for both the assembly’s spontaneity and the interfacial coupling inferred from spectroscopy. The ZIF-8/Ag_3_PO_4_ heterostructure was constructed by placing the ZIF-8 cluster above the Ag_3_PO_4_(110) surface at an initial separation of 3 Å. Several initial configurations were generated by rotating the MOF about the surface normal (*z*-axis) in 30° increments, thereby exploring distinct interfacial arrangements and adsorption geometries. The adsorption energies, calculated using eq 1, range from −4.56 to −8.78 eV. The relatively broad variation in adsorption energy reflects the strong dependence of the interfacial stabilization on the relative orientation of the ZIF-8 cluster. Rotation changes the identity and number of atoms involved in close interfacial contacts and consequently modifies the balance between dispersion, electrostatic interactions, charge redistribution, and specific chemical interactions at the interface. The optimized geometries for all configurations are shown in Figure S1 of the SM. The negative adsorption energies indicate that the interaction between Ag_3_PO_4_ and the MOF is thermodynamically favorable, supporting spontaneous heterostructure formation. The system with the most negative adsorption energy (zero-degree rotation and *E*_ads_ of −8.78 eV) was chosen for further analysis and discussion. After full geometric optimization, the ZIF-8/Ag_3_PO_4_ system adopts a stable interfacial configuration characterized by significant structural rearrangements in the contact region. Structural relaxation promotes subtle atomic displacements that enhance interfacial stabilization and minimize the system’s total energy. The optimized geometries reveal that ZIF-8 adsorption significantly modifies the local coordination environment of Ag and O atoms at the Ag_3_PO_4_ surface. In particular, the Ag-centered coordination polyhedra located near the interface become distorted upon adsorption, reflecting the establishment of specific interactions between the semiconductor surface and the MOF linker groups. These distortions are accompanied by variations in Ag–O and P–O bond lengths, predominantly in the interfacial region, indicating that adsorption strongly influences the semiconductor’s local structure. The largest structural changes occur in the surface Ag atoms directly involved in the interaction with the MOF, hereafter denoted as Ag1, Ag2, and Ag3 (Figure 3). These calculated distortions provide a direct structural counterpart to the defect-sensitive changes detected experimentally by PL and FTIR.

**Figure 3:**
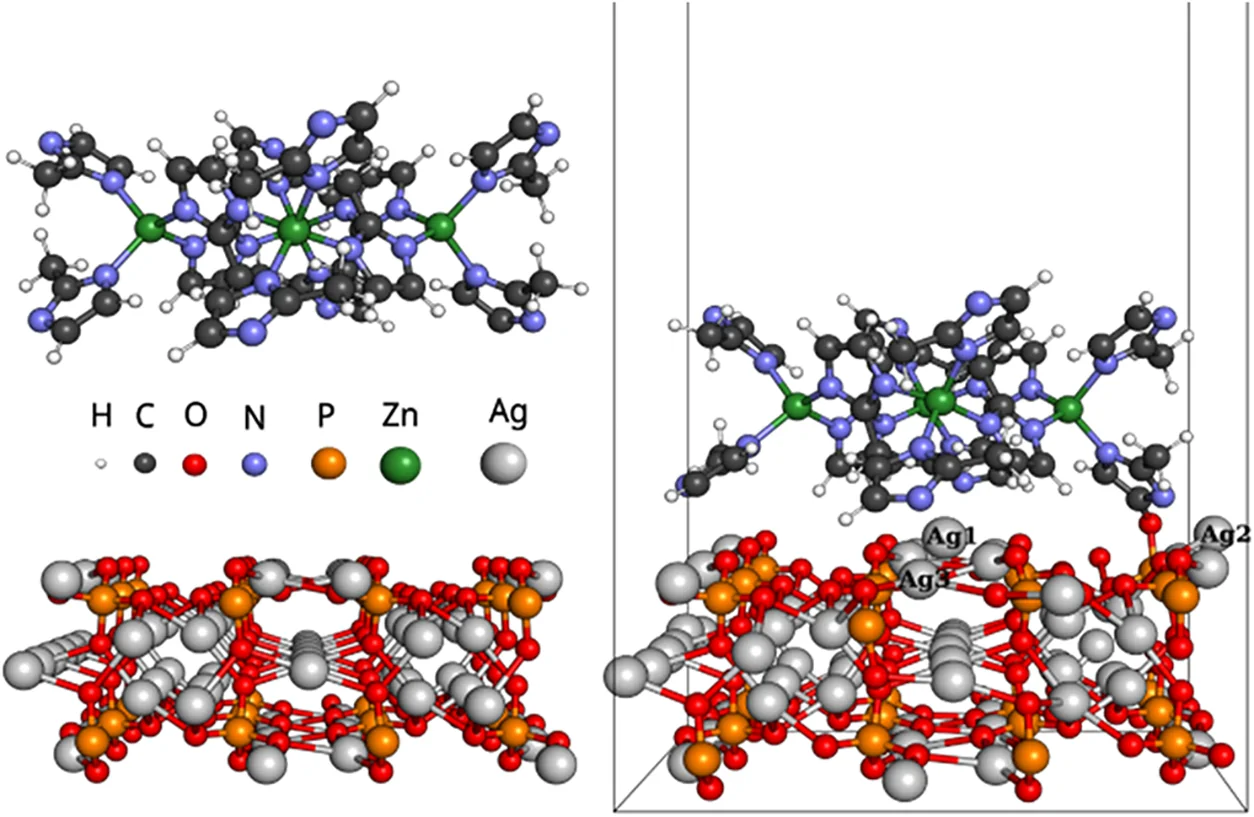
Optimized geometries of MOF, Ag_3_PO_4_, and the ZIF-8/Ag_3_PO_4_ heterostructure. The Ag atoms in the more distorted polyhedra are highlighted.

Field-emission scanning electron microscopy (FE-SEM) micrographs in Figure 4A–C reveal the morphological characteristics of the pristine materials and the resulting heterostructure. Ag_3_PO_4_ exhibits irregular polyhedral-like particles with relatively compact agglomerates, whereas ZIF-8 presents its characteristic rhombic dodecahedral morphology with smaller particle dimensions and rougher surface texture. In the ZIF-8/Ag_3_PO_4_ heterostructure, both morphologies coexist in intimate contact, clearly demonstrating successful interfacial assembly between the two components. The FE-SEM images suggest that ZIF-8 particles are homogeneously distributed across the Ag_3_PO_4_ surface, thereby creating a highly interconnected interfacial region. This intimate interfacial contact is particularly important because it maximizes the heterojunction area available for charge transfer processes. The close coupling between ZIF-8 and Ag_3_PO_4_ likely facilitates rapid carrier migration across the interface while simultaneously increasing the density of catalytically active surface sites. Additionally, the porous nature of ZIF-8 may enhance adsorption capacity and increase local reactant concentration near the semiconductor surface, thereby further improving interfacial reaction kinetics. It is important to note that previous studies have confirmed no Ag NP evolution in the ZIF-8 particles if they are not in contact with a silver-based semiconductor.^28^ Analogously, Ag^+^ reduction in Ag_3_PO_4_ to form Ag NPs has been extensively investigated.^23,34^

**Figure 4:**
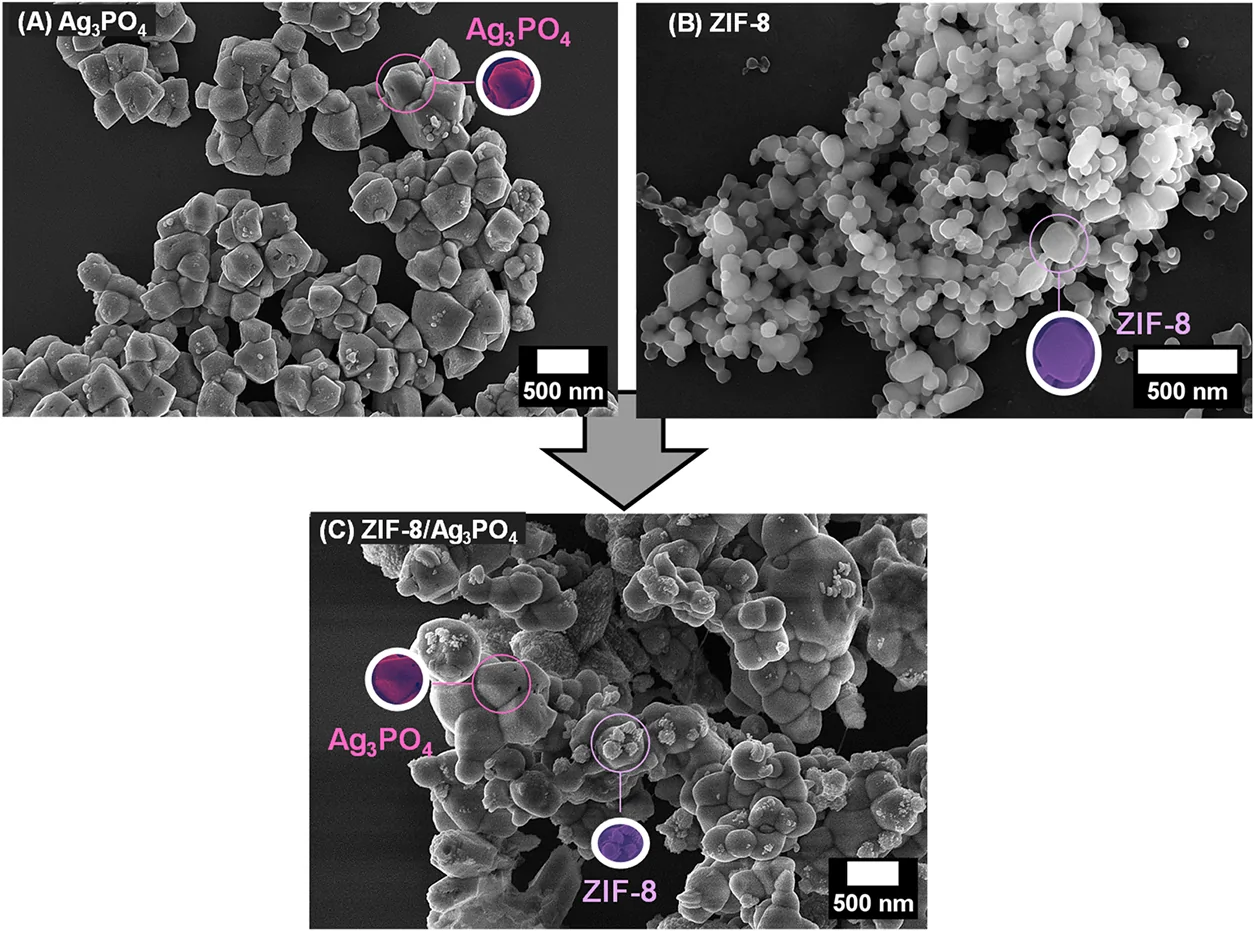
SEM micrograph images for the samples (A) Ag_3_PO_4_, (B) ZIF-8, and (C) ZIF-8/Ag_3_PO_4_ heterostructure, respectively.

To further probe the electronic effects associated with heterostructure formation, Bader charge analyses were performed. The results reveal a net charge transfer of approximately 0.15 e from the Ag_3_PO_4_ surface to the ZIF-8, indicating the formation of an intrinsic interfacial electric field. This charge redistribution is expected to facilitate charge separation and promote electronic communication between the two components. Notably, charge transfer is not uniformly distributed across the interface. The largest charge variations occur on undercoordinated Ag atoms in the outermost surface layers, indicating that these sites serve as preferential interaction centers for the ZIF-8′s imidazolate groups. Likewise, surface oxygen atoms directly involved in interfacial contacts show significant changes in their effective charges, further confirming strong electronic coupling between the semiconductor and the framework. The electron density difference maps in Figure 5 clearly visualize these effects, revealing regions of charge accumulation and depletion associated with the specific interactions between the Ag_3_PO_4_ surface and the ZIF-8 linker groups.

**Figure 5:**
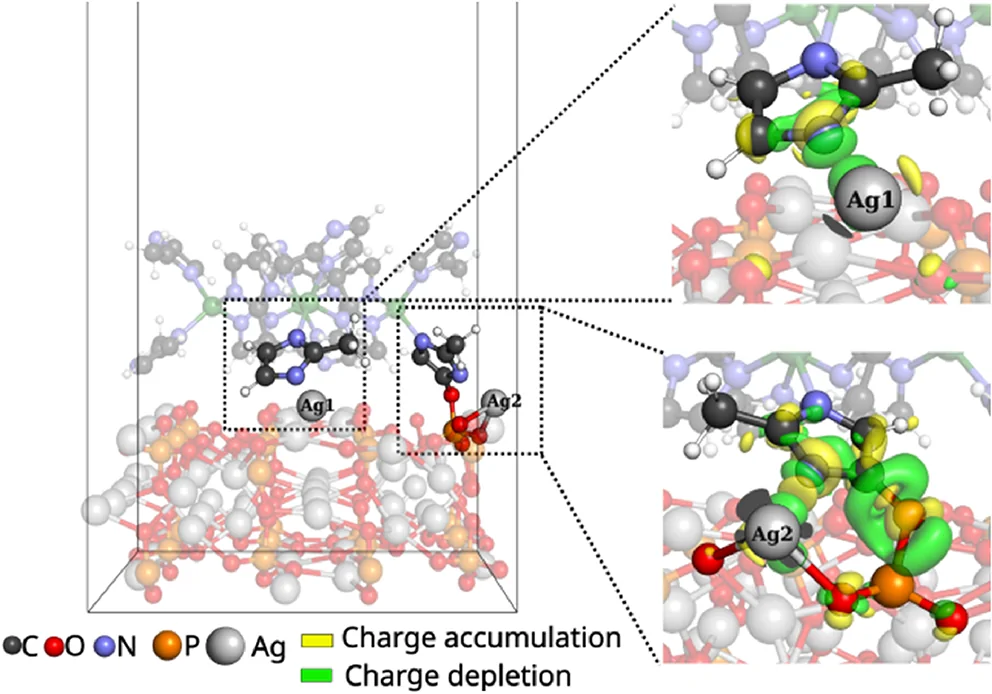
Optimized ZIF-8/Ag_3_PO_4_ heterostructure. A zoomed-in electron density difference map shows electron density transfer from the Ag2 atom to an N atom in the imidazolate group and the formation of a bond between a surface O atom and a C atom in the same MOF linker group.

Having established a robust electronic coupling at equilibrium, we next investigate the heterostructure’s response to electron irradiation, revealing dynamic behavior faithfully reproduced by first-principles calculations. Figure 6A–F presents a time-resolved SEM investigation of the ZIF-8/Ag_3_PO_4_ heterostructure under continuous electron-beam irradiation, revealing the dynamic nucleation and growth of metallic Ag NPs on the ZIF-8 surface, consistent with the redistribution of Ag species from the Ag_3_PO_4_ component toward ZIF-8. The sequential micrograph images recorded from 0 to 5 min clearly show that electron irradiation induces progressive structural and electronic rearrangements that culminate in the selective formation and accumulation of Ag NPs preferentially over the ZIF-8 domains rather than uniformly across the Ag_3_PO_4_ surface. At the initial irradiation stage (*t* = 0 min), the heterostructure exhibits well-defined interfaces between the larger Ag_3_PO_4_ particles and the smaller ZIF-8 crystallites, indicating that upon irradiation, Ag NP formation occurs readily. As irradiation proceeds (*t* = 1–2 min), brighter nanoscale contrast regions emerge near the interfacial zones, indicating the initial reduction of Ag^+^ species and the nucleation of metallic Ag clusters. Interestingly, these early stage Ag nuclei appear spatially correlated with regions containing ZIF-8 particles, suggesting that the heterointerface plays a decisive role in directing electron accumulation and subsequent Ag reduction. With increasing irradiation time (*t* = 3–5 min), a pronounced migration and coalescence process is observed, leading to larger Ag NP agglomerates predominantly localized on the ZIF-8 surface. The dashed regions highlighted in the SEM images emphasize the progressive redistribution of material across the interface, strongly suggesting that the electron beam induces directional diffusion of Ag species from Ag_3_PO_4_ toward the ZIF-8 domains. This behavior is consistent with irradiation-induced reduction processes commonly observed in Ag-based semiconductors, in which incident electrons promote local electronic excitation, bond destabilization, and the release of mobile Ag species.^28,29,35–39^ Complementarily, Figure S2A–F and Figure S2G–L show the evolution of Ag NPs over the Ag_3_PO_4_ and ZIF-8 particles, respectively. It is also important to know that Ag NP growth on the ZIF-8 surface is not necessarily gradual, as the surface may become saturated.

**Figure 6:**
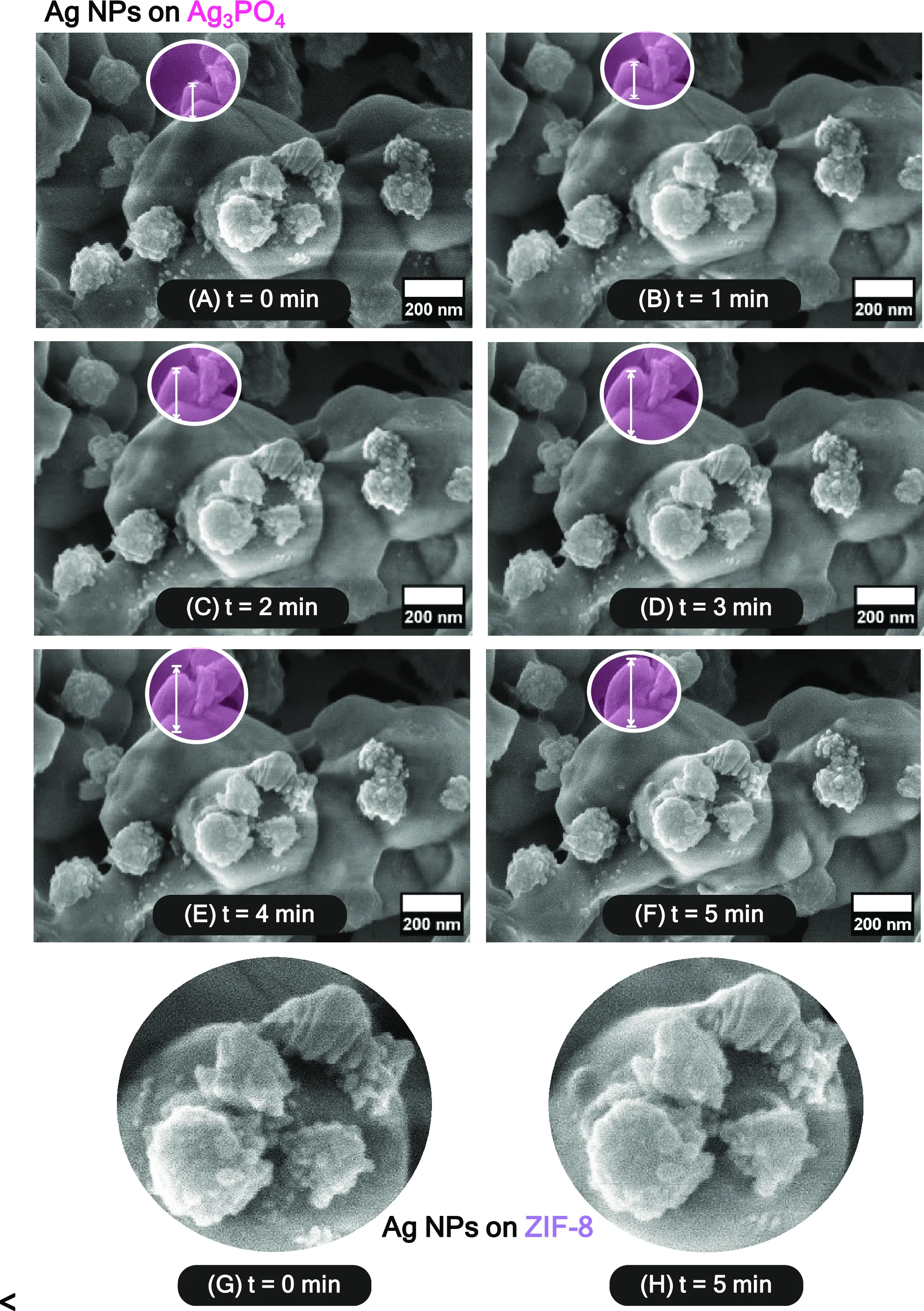
(A–F) SEM micrograph images obtained of the ZIF-8/Ag_3_PO_4_ heterostructure, highlighting the selective growth and agglomeration of Ag NPs on top of the ZIF-8 particles. Time-lapse micrographs were recorded at 1 min intervals over 5 min. The insets (G, H) show the evolution of Ag NPs atop the ZIF-8 particles over 5 min of EBI.

The preferential accumulation of Ag NPs on ZIF-8 indicates that the MOF acts as an energetically favorable trapping and stabilization platform for reduced Ag atoms. Because of its highly porous structure, large surface area, and abundant coordination sites associated with nitrogen-containing imidazolate linkers, ZIF-8 likely provides low-energy adsorption sites that stabilize migrating Ag species and suppress their reoxidation. Simultaneously, the heterojunction formed between Ag_3_PO_4_ and ZIF-8 may establish localized interfacial electric fields that promote directional electron transfer under irradiation, further facilitating selective Ag^+^ reduction near the MOF’s surface. From a mechanistic perspective, the observations strongly suggest that electron irradiation triggers a sequence involving: (i) local electronic excitation and partial structural destabilization of Ag_3_PO_4_, (ii) reduction of Ag^+^ into metallic Ag^0^ species, (iii) interfacial diffusion of reduced Ag atoms/clusters, and finally (iv) preferential nucleation and agglomeration over ZIF-8 domains. The gradual increase in nanoparticle size over time further indicates that the process proceeds via diffusion-limited growth and nanoparticle coalescence rather than isolated nucleation events. Interestingly, electron-beam irradiation also revealed the progressive decrease and eventual disappearance of pre-existing Ag NPs at the Ag_3_PO_4_ surface (Figure S3A–C and Video S1). This behavior is consistent with the redistribution of Ag species into the Ag_3_PO_4_ phase and suggests that electron-beam-induced Ag evolution is a dynamic process involving not only Ag^+^ reduction, redistribution, nucleation, and growth, but also the reverse incorporation of Ag species into the semiconductor. This behavior highlights the dynamic nature of the Ag/Ag_3_PO_4_ system under electron irradiation, where the local structural and electronic environment may continuously modulate the stability and spatial distribution of reduced Ag species.

The effects of electron irradiation were investigated using DFT calculations by progressively adding excess electrons to the ZIF-8/Ag_3_PO_4_ heterostructure. Electron injection induces significant structural and electronic modifications that become increasingly pronounced as the number of added electrons increases from 1 to 5. Figure S4 shows the optimized ZIF-8/Ag_3_PO_4_ heterostructures for different excess electron numbers, and Table 1 summarizes the Bader charge analysis for these systems. The results show that injected electrons distribute between both components of the hybrid system, indicating that ZIF-8 actively participates in charge accommodation rather than acting solely as an inert support. For low electron concentrations, the excess charge is almost equally partitioned between the MOF and Ag_3_PO_4_. However, as the number of added electrons increases, Ag_3_PO_4_ becomes the preferential electron reservoir, accommodating about 56% of the injected charge with five additional electrons.

**Table 1:** Bader Charge Analysis of the ZIF-8/Ag_3_PO_4_ Heterostructure as a Function of the Number of Added Electrons

Added e^–^	ZIF-8 (e)	Ag_3_PO_4_ (e)	Total (e)	Δ*q* (ZIF-8) (e)^a^	Δ*q* (Ag_3_PO_4_) (e)^a^	ZIF-8 (%)^b^	Ag_3_PO_4_ (%)^b^
**0**	–0.15	+0.15	0.00	0.00	0.00		
**1**	–0.63	–0.37	–1.00	–0.48	–0.53	47.5	52.5
**2**	–1.15	–0.85	–2.00	–1.00	–1.00	50.0	50.0
**3**	–1.60	–1.40	–3.00	–1.45	–1.55	48.3	51.7
**4**	–1.92	–2.08	–4.00	–1.77	–2.23	44.3	55.7^a^
**5**	–2.35	–2.65	–5.00	–2.20	–2.81^b^	43.9	56.1

a Charge accumulated relative to the relaxed heterostructure without added electrons (0e).

b Fraction of the injected electronic charge accommodated by each component, calculated from the relative Bader charge variation with respect to the pristine heterostructure.

This trend suggests that the MOF’s low-energy acceptor states fill progressively, while additional electrons increasingly populate states localized within the Ag_3_PO_4_ phase. The preferential charge accumulation within Ag_3_PO_4_ is consistent with the progressive reduction of surface Ag cations and the structural distortions observed in the optimized geometries. In particular, the Ag atoms located at the heterointerface (Ag1, Ag2, and Ag3) exhibit the largest structural changes upon electron injection, indicating that these sites act as preferential electron-accumulation centers.

To investigate the dynamic consequences of charge accumulation, *ab initio* molecular dynamics (AIMD) simulations were performed. The optimized heterostructure with four added electrons was used as the initial configuration and subjected to a sequential thermal protocol consisting of 100 steps at 50 K, 150 steps at 150 K, and 500 steps at 300 K. The final AIMD configuration is shown in Figure 7.

**Figure 7:**
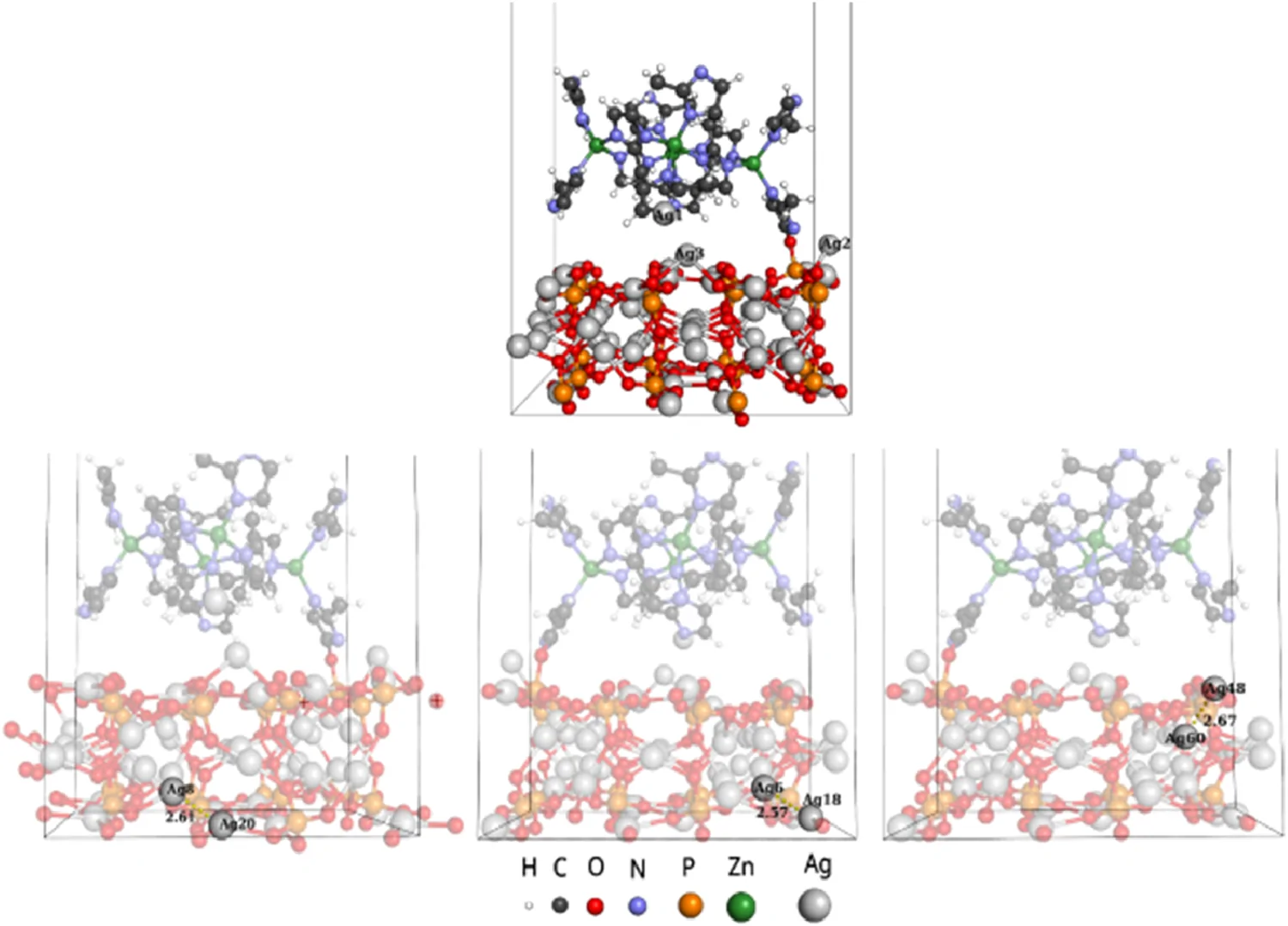
Final AIMD configuration of the ZIF-8/Ag_3_PO_4_ heterostructure containing four excess electrons. The migration of interfacial Ag atoms toward the MOF and the formation of three Ag dimers within the Ag_3_PO_4_ framework are highlighted.

The AIMD simulations demonstrate that the structural trends identified in the static calculations are further amplified under finite-temperature conditions. The interfacial Ag atoms become increasingly mobile, leading to substantial rearrangements at the semiconductor/MOF interface. Most notably, Ag3 becomes completely detached from the Ag_3_PO_4_ surface and establishes interactions with the MOF framework. Ag1 and Ag2 also exhibit stronger interactions with neighboring linker groups, indicating that electron-rich conditions promote the migration of surface Ag species toward the interface.

Significant structural modifications are also observed within the semiconductor region. Inspection of the final AIMD structure reveals the formation of three Ag–Ag dimers within the Ag_3_PO_4_ lattice, indicating the onset of Ag aggregation processes. The emergence of these Ag-rich motifs is consistent with the preferential localization of excess charge within the semiconductor and supports the hypothesis that electron irradiation promotes the reduction and migration of Ag species. Taken together, the Bader charge analysis and AIMD simulations suggest that the ZIF-8/Ag_3_PO_4_ heterostructure evolves under electron-rich conditions via coupled charge transfer and structural reconstruction, ultimately favoring the formation of Ag-rich domains at the heterointerface.

A detailed analysis of the AIMD trajectories shows that electron-rich conditions promote significant silver mobility within the Ag_3_PO_4_ lattice. As the simulation progresses, several surface and subsurface Ag atoms undergo collective displacements that progressively reduce Ag–Ag distances. Consequently, three Ag dimers form in the semiconductor region, as shown in Figure 7. The emergence of these Ag-rich species is accompanied by distortions of the surrounding Ag–O coordination environments, indicating partial destabilization of the original Ag_3_PO_4_ framework. The formation of Ag dimers is consistent with Bader charge analysis, which shows preferential accumulation of excess electrons within the Ag_3_PO_4_ phase, facilitating Ag^+^ reduction and strengthening Ag–Ag interactions. The formation of three Ag dimers indicates that the electron-induced structural reconstruction is not restricted to the heterointerface but also propagates into the semiconductor bulk, where reduced Ag species become sufficiently mobile to aggregate. These results suggest that electron injection triggers the initial stages of silver aggregation, a precursor process to the nucleation of larger metallic Ag clusters under irradiation conditions.

Moreover, the atomic mobility induced by electron injection, the mean-square displacement (MSD), was calculated from the AIMD trajectories, as shown in Figure 8. The MSD curves increase continuously throughout the simulation for all atomic species, indicating progressive structural rearrangement of the heterostructure under electron-rich conditions. However, the magnitude and evolution of atomic displacements depend strongly on the chemical species.

**Figure 8:**
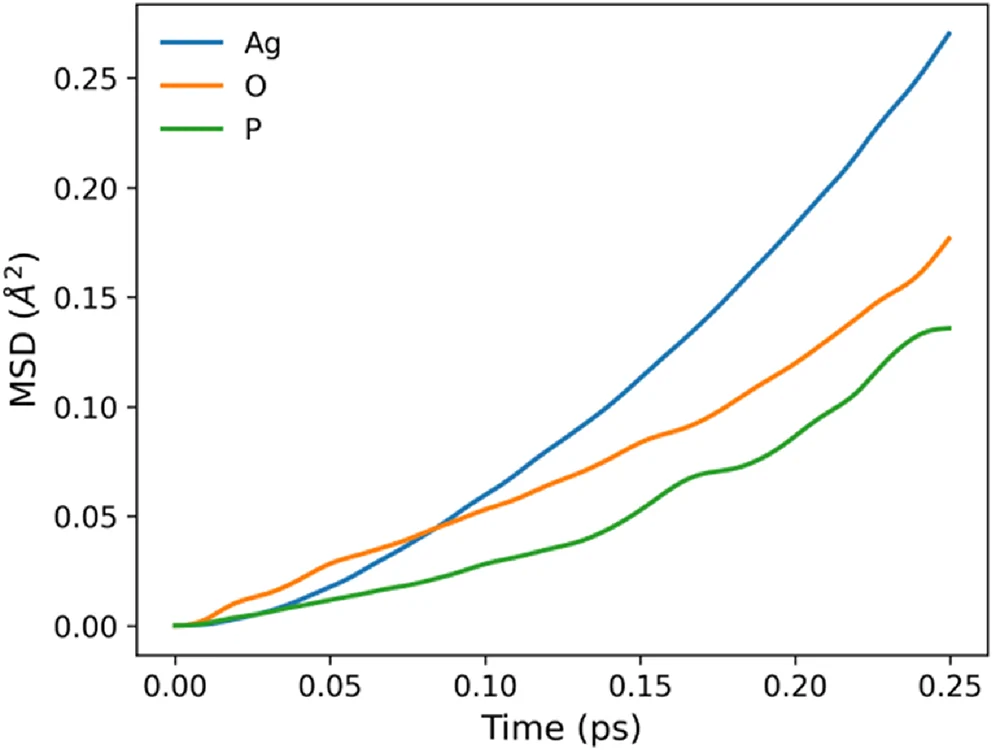
Mean square displacement (MSD) profiles of Ag, O, and P atoms during AIMD simulations of the ZIF-8/Ag_3_PO_4_ heterostructure containing excess electrons. The higher MSD values for Ag indicate greater silver mobility than for the phosphate framework atoms.

During the initial stages of the simulation, oxygen atoms show slightly larger MSD values than silver atoms, reflecting rapid relaxation of the Ag–O framework immediately after electron injection. As the simulation progresses, however, the MSD of Ag increases more rapidly and surpasses those of both O and P after approximately 0.08 ps. At the end of the trajectory, Ag exhibits the largest MSD value, reaching approximately 0.27 Å^2^, whereas O and P attain values of about 0.18 and 0.14 Å^2^, respectively. These results demonstrate that silver atoms become the most mobile species in the irradiated heterostructure.

The end point of this process, the selective nucleation of metallic silver, is directly evidenced by HAADF-STEM imaging combined with elemental mapping and local EDS analysis after irradiation. Figure 9A–C displays HAADF-STEM imaging combined with elemental mapping and local EDS analyses of the ZIF-8/Ag_3_PO_4_ heterostructure after electron irradiation, providing direct evidence for the selective formation and localization of Ag NPs at the heterointerface. The HAADF-STEM image in Figure 9A reveals two chemically distinct regions corresponding to Ag_3_PO_4_ and ZIF-8, while the highlighted areas identify the specific regions selected for local compositional analysis. The EDS spectra collected from Areas 1 and 2 confirm the coexistence of the two components in the heterostructure. Area 1 exhibits intense Ag, P, and O signals characteristic of Ag_3_PO_4_, whereas Area 2 shows dominant Zn, C, and N contributions associated with the ZIF-8 framework. Areas 3–5, corresponding to nanoparticle-like features within the ZIF-8-rich region, show Ag and Zn signals along with strongly suppressed P contributions compared with the Ag_3_PO_4_-rich region (Area 1). These results indicate the formation of Ag-rich, phosphate-depleted nanodomains associated with the ZIF-8 phase after electron irradiation. A weak residual P contribution can still be detected in some regions, particularly Area 4, which may arise from the neighboring phosphate-containing phase within the nanoscale EDS sampling volume. Since EDS does not provide information on the Ag oxidation state, these measurements alone cannot distinguish Ag^+^ from Ag^0^. The assignment of these Ag-rich domains to reduced Ag species is therefore made in conjunction with the established electron-beam-induced Ag^+^-to-Ag^0^ conversion in Ag_3_PO_4_ reported previously. HRTEM analysis (Figure S5A–C) provides further structural evidence, revealing well-resolved lattice fringes with an interplanar spacing of 0.238 nm in a nanoparticle at the ZIF-8 interface, consistent with the (111) planes of face-centered cubic metallic Ag. This observation provides direct nanoscale evidence supporting the formation of Ag0 and its presence at the ZIF-8/Ag_3_PO_4_ interface.

**Figure 9:**
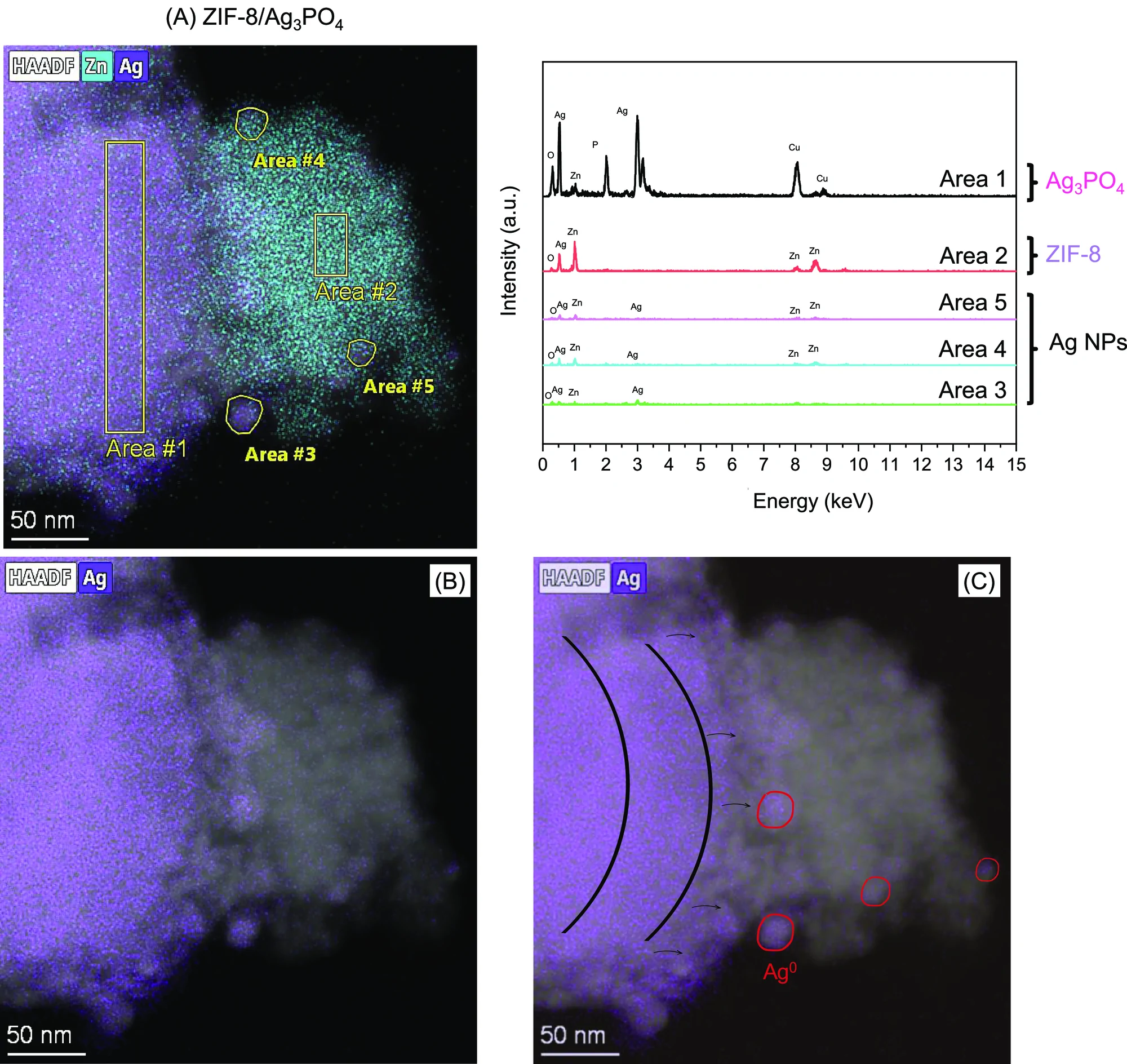
(A–C) Point-by-point EDS analysis and elemental maps obtained for different regions in the ZIF-8/Ag_3_PO_4_ heterostructure via TEM measurements. The results highlight the selective growth and agglomeration of Ag NPs atop the ZIF-8 particles.

The enhanced mobility of Ag atoms is fully consistent with the structural evolution observed during AIMD, particularly the displacement of the interfacial Ag atoms (Ag1–Ag3) and the formation of three Ag–Ag dimers within the Ag_3_PO_4_ lattice. Moreover, the Bader charge analysis revealed that excess electrons accumulate preferentially within the Ag_3_PO_4_ component of the heterostructure. This charge accumulation is expected to promote partial reduction of Ag^+^ centers, weaken local Ag–O interactions, and facilitate Ag migration through the lattice.

Notably, the significantly larger MSD values observed for Ag compared with O and P indicate that electron injection induces a selective enhancement of silver mobility rather than merely promoting local lattice distortions. If the excess electrons only generated vibrational relaxation of the phosphate framework, the dominant atomic displacements would be expected to originate primarily from oxygen atoms and from distortions of the PO_4_ tetrahedra. Instead, the sustained growth of the Ag MSD demonstrates that silver atoms become the most mobile species in the system under electron-rich conditions.

The enhanced Ag mobility is directly reflected in the structural evolution observed during AIMD, particularly through the formation of three Ag–Ag dimers within the Ag_3_PO_4_ lattice. To further elucidate the electronic nature of these Ag-rich species, Bader charges were analyzed for the Ag atoms involved in dimer formation (Table 2). The results reveal a highly heterogeneous charge redistribution among the three dimers. In particular, the Ag6–Ag18 dimer exhibits the largest net charge accumulation, with a decrease of approximately 0.42 e in its combined Bader charge between the neutral and the 5e systems, indicating preferential localization of excess electrons within this Ag-rich region. A similar, although less pronounced, behavior is observed for the Ag48–Ag60 dimer. In contrast, the Ag8–Ag20 pair shows no net electron accumulation, suggesting that its formation is driven predominantly by structural reconstruction rather than direct charge localization.

**Table 2:** Bader Charges (e) of the Ag–Ag Dimers Formed during the AIMD Simulation as a Function of the Number of Added Electrons

Dimer	0e	1e	2e	3e	4e	5e	Δ*q* (0e→5e)
Ag6–Ag18	1.814	1.790	1.539	1.275	1.318	1.398	–0.416
Ag8–Ag20	1.035	0.923	1.392	1.522	1.837	1.604	+0.569
Ag48–Ag60	1.571	1.538	1.456	1.288	1.493	1.476	–0.095

These results demonstrate that the injected electrons do not distribute uniformly throughout the Ag_3_PO_4_ lattice but instead localize preferentially at specific Ag centers, which subsequently become active sites for structural rearrangement and Ag–Ag bond formation. The coexistence of electronically enriched and structurally driven dimers indicates that charge localization and atomic migration occur simultaneously during electron-induced reconstruction. Combined with the preferential accumulation of excess charge in the Ag_3_PO_4_ component, the increasing Ag mobility revealed by the MSD analysis, and the direct observation of Ag aggregation during AIMD, these findings strongly support a mechanism in which electron accumulation promotes the reduction of selected Ag^+^ centers, facilitates silver diffusion through the lattice, and ultimately triggers the initial stages of metallic silver nucleation within the ZIF-8/Ag_3_PO_4_ heterostructure.

Theoretical and experimental results indicate a strong interaction between Ag_3_PO_4_ and ZIF-8, leading to heterostructure formation. The flexibility of the Zn–N bonds enables “gate-opening” effects, in which the pore structure adapts to accommodate silver clusters. ZIF-8 acts as a stable, hierarchical matrix that hosts and disperses Ag NPs, which are efficient electron collectors due to the collective oscillation of electrons on the metal surface. The framework prevents these species from becoming disordered, which would otherwise reduce electron-transfer efficiency to semiconductors. Thus, when the ZIF-8/Ag_3_PO_4_ system is irradiated with high-energy electrons, it promotes electrons from lower to higher energy states. In ZIF-8, electrons are excited from the valence band (VB) to a state too close to the conduction band (CB), the forbidden region. In this way, ZIF-8 plays a crucial role in promoting confined nanoscale pores and defect states. These pores have potential wells due to localized pi-electron resonance in the imidazole ring. The electron wave function becomes spatially localized due to the MOF’s potential landscape. This reduces recombination probability and creates a probabilistic condition for electrons to transfer to the silver phosphate semiconductor. This electron-transfer process is governed by the overlap between the ZIF-8 conduction-band donor state and the acceptor state of the [AgO_6_] clusters.

## Conclusion

By combining time-resolved HRTEM observations with DFT and *ab initio* molecular dynamics simulations, this work elucidates the atomistic mechanism governing electron-induced silver transport in the ZIF-8/Ag_3_PO_4_ heterostructure. Electron irradiation reduces Ag^+^ species within the Ag_3_PO_4_ lattice, generating highly mobile Ag atoms that migrate toward the heterointerface and progressively aggregate into metallic Ag NPs. Theoretical calculations reveal that excess electrons accumulate preferentially in the Ag_3_PO_4_ component, thereby enhancing the mobility of reduced Ag species and promoting the formation of Ag–Ag dimers that mark the initial stages of metallic silver nucleation. Beyond serving as a structural support, ZIF-8 actively participates in interfacial processes by accepting charge from Ag_3_PO_4_, providing energetically favorable pathways for Ag migration, and stabilizing the resulting metallic NPs. Under prolonged electron-beam irradiation, the initially formed Ag nanoparticles undergo further dynamic redistribution toward the Ag_3_PO_4_ region and eventually become incorporated within the irradiated semiconductor region. This observation is morphological in nature and does not imply reoxidation of Ag^0^ to Ag^+^ or restoration of the original Ag_3_PO_4_ crystal lattice. These findings reveal a previously unexplored mechanism of reversible interfacial mass transport driven by coupled charge transfer, Ag reduction, atomic diffusion, and structural rearrangement. This study shows that electron irradiation is a versatile technique for creating dynamic semiconductor/MOF heterostructures at the atomic level. Triggering and controlling reversible metal migration across hybrid interfaces paves the way for adaptable photocatalysts, plasmonic materials, and multifunctional porous heterostructures with tunable electronic and catalytic properties.

## Supplementary Material





## Data Availability

Data will be made available on request.

## Notes

Additional characterization data (i.e., density functional theory (DFT) calculations) (PDF)
The progressive decrease and eventual disappearance of pre-existing Ag NPs at the Ag_3_PO_4_ surface (MP4)
