# Supplementary material for: Electron-Beam-Driven Ag Nanoparticle Evolution at ZIF-8/Ag3PO4 Heterointerfaces
Source: Langmuir. 2026 Sep 24;42(39):29371–83. doi: 10.1021/acs.langmuir.6c04357 (PMC13648263; doi:10.1021/acs.langmuir.6c04357)
Supplement: Supplementary file 1 [file la6c04357_si_001.pdf]

Supporting Information

Electron-Beam-Driven Ag Nanoparticle Evolution at ZIF-8/Ag_3PO_4 Heterointerfaces

Júlia T. Ichikura[a], Gustavo H. F. Candido[b], Murilo Nader[a], Giovanna A. Grasser[a], Márcio D. Teodoro[c], Elson Longo[a], Henrique Moreno[a,*], Miguel A. San-Miguel[b].

[a] CDMF-LIEC,Federal University of São Carlos (UFSCar), São Carlos, 13565-905, Brazil.
[b] Institute of Chemistry, Universidade Estadual de Campinas (UNICAMP), Campinas, 13083-970, Brazil.
[c] Department of Physics, Federal University of São Carlos, São Carlos, SP, 13565-905, Brazil.
*corresponding author: hmoreno@ufscar.br

Table of Contents

Figure S1. Optimized ZIF-8/Ag_3PO_4 heterostructures corresponding to different initial rotational orientations of the ZIF-8 cluster and the corresponding adsorption energies ………………………………………………………………… S2

Figure S2. Ag NP distribution on the (A-F) Ag_3PO_4 and (G-L) ZIF-8 surfaces under continuous electron-beam irradiation, shown at 1 min intervals over 5 min ……………………………………………………………………………………… S2

Figure S3. Time-resolved SEM images acquired under continuous electron-beam irradiation showing the progressive evolution of a metallic Ag nanoparticle (Ag0) at the Ag3PO4 surface after (B) 5 s, (C) 15 s, and (D) 30 s of irradiation, showing Ag^0's reincorporation into the Ag3PO4 structure ………………………………………… S3

Figure S4. Optimized ZIF-8/Ag3PO4 heterostructures corresponding to different excess electrons …………… S3

Figure S5. HRTEM analysis showing the ZIF-8/Ag_3PO_4 interface and the presence of an Ag NPs at the interfacial region. (B) Magnified HRTEM image of the selected Ag nanoparticle, revealing well-resolved lattice fringes. (C) Intensity profile obtained from the selected region in (B), showing an interplanar spacing of 0.238 nm, consistent with the (111) planes of face-centered cubic metallic Ag (Ag^0)………………………………………………… S4

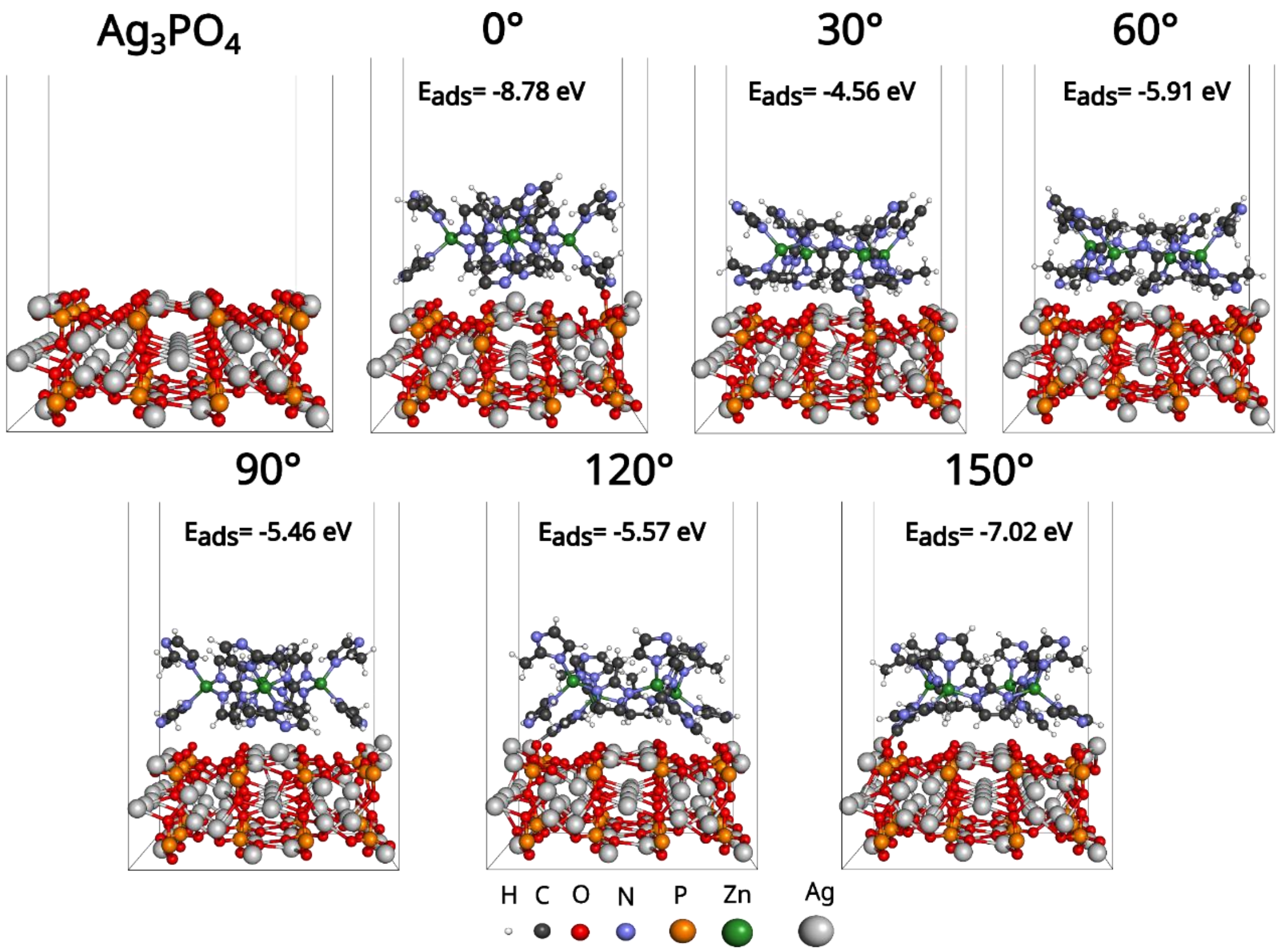

Figure S1. Optimized ZIF-8/Ag_3PO_4 heterostructures corresponding to different initial rotational orientations of the ZIF-8 cluster and the corresponding adsorption energies.

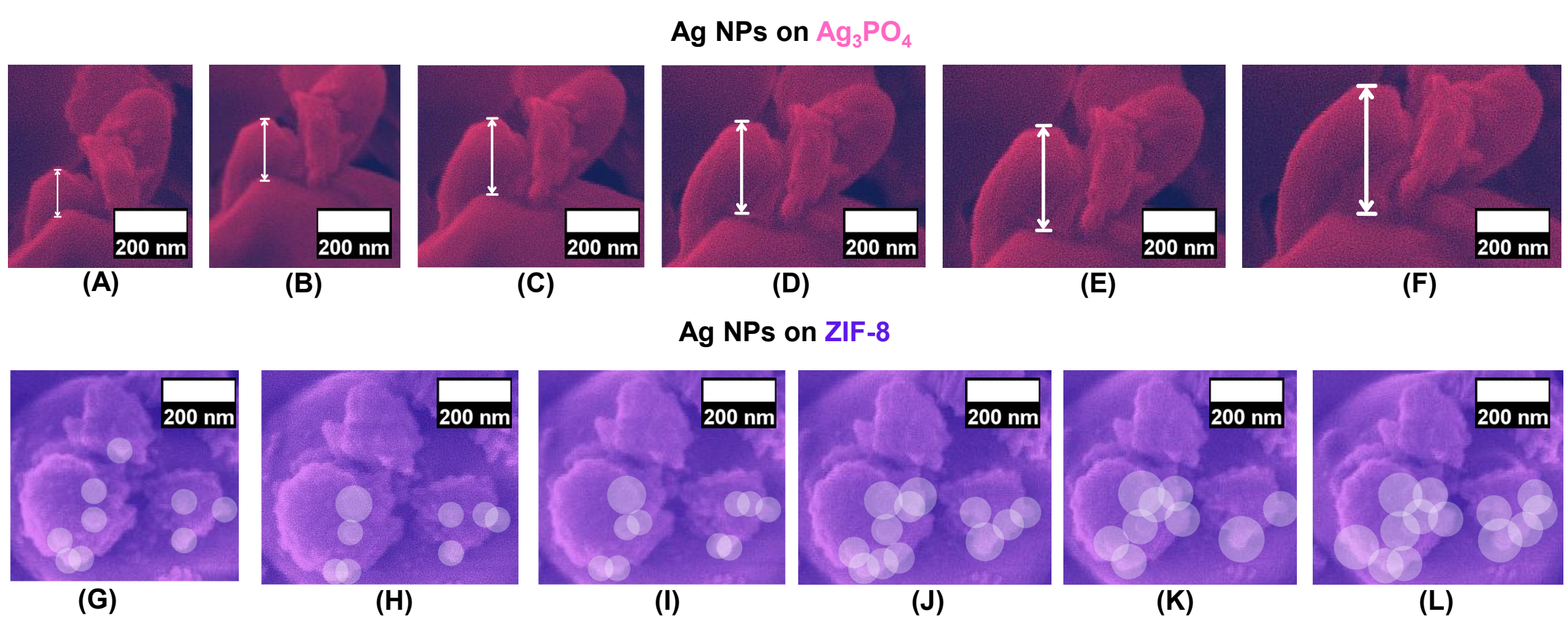

Figure S2. Ag NP distribution on the (A-F) Ag_3PO_4 and (G-L) ZIF-8 surfaces under continuous electron-beam irradiation, shown at 1 min intervals over 5 min.

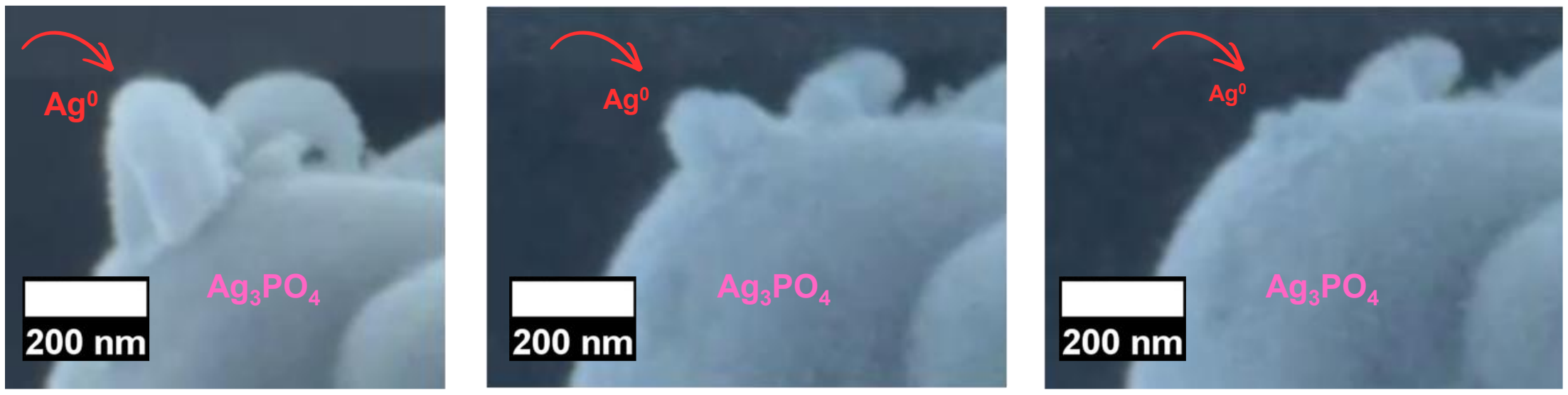

(A) 5 s (B) 15 s (C) 30 s

Figure S3. Time-resolved SEM images acquired under continuous electron-beam irradiation showing the progressive evolution of a metallic Ag nanoparticle (Ag^0) at the Ag_3PO_4 surface after (B) 5 s, (C) 15 s, and (D) 30 s of irradiation, showing Ag^0's reincorporation into the Ag_3PO_4 structure.

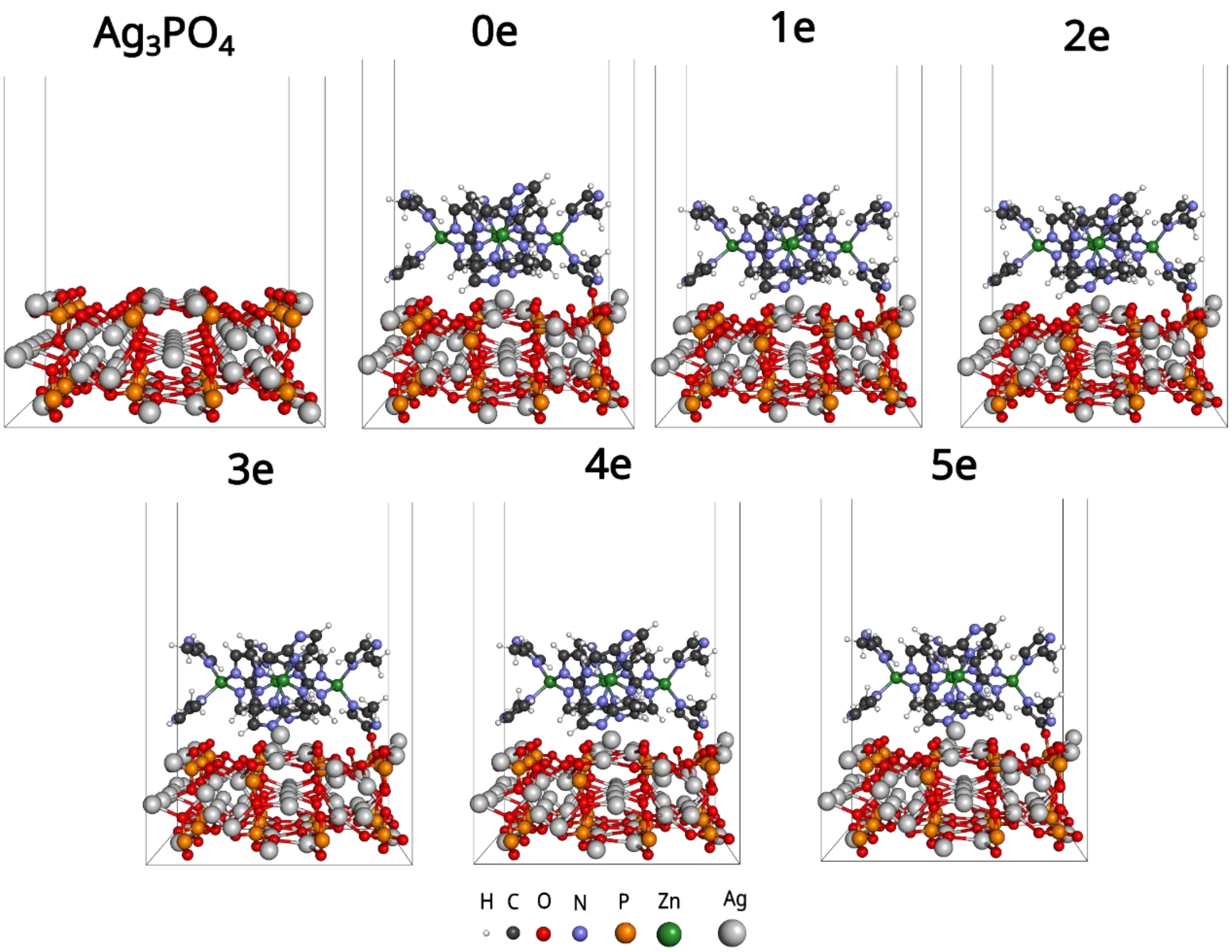

Figure S4. Optimized ZIF-8/Ag_3PO_4 heterostructures corresponding to different excess electrons.

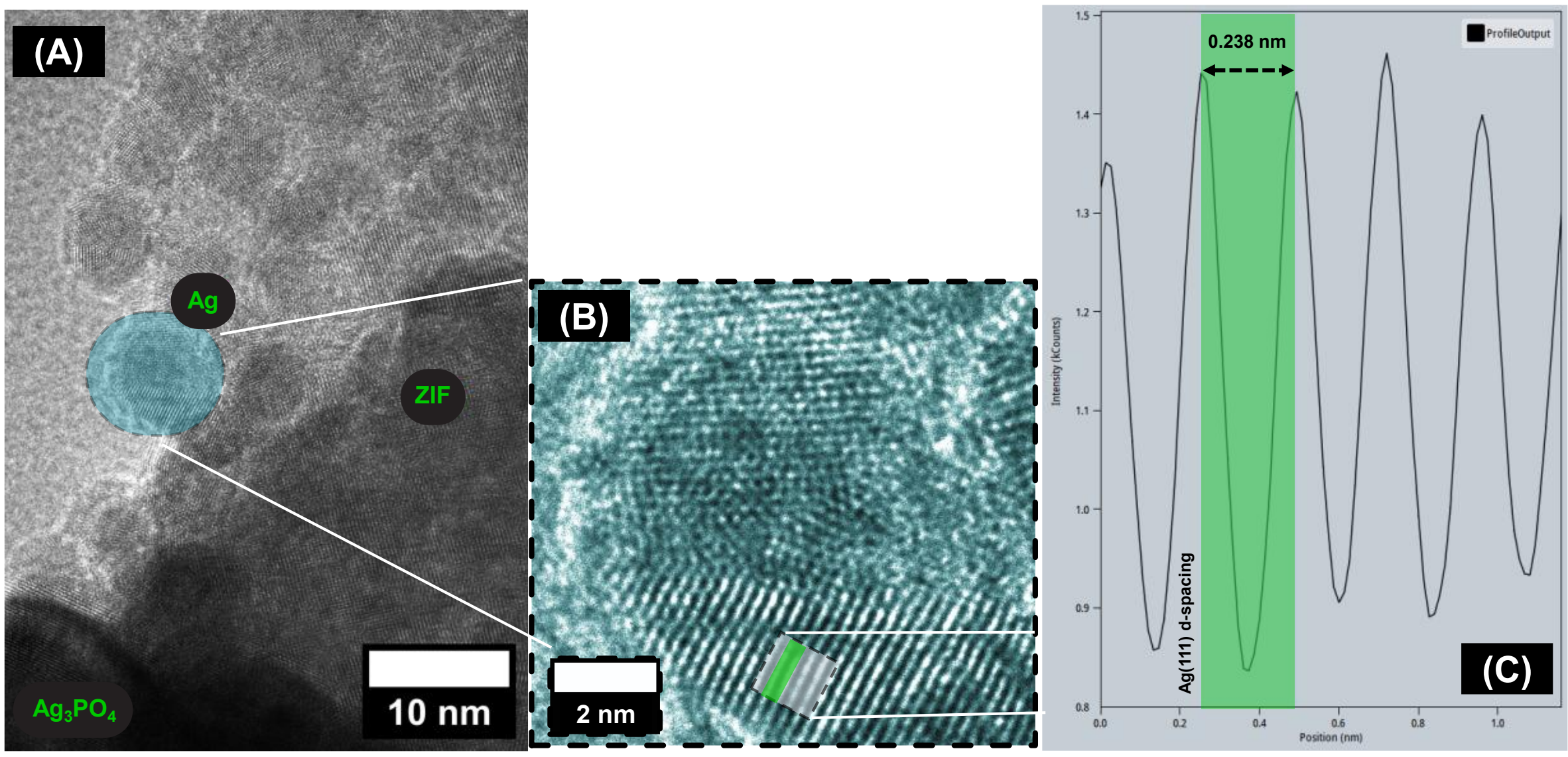

Figure S5. HRTEM analysis showing the ZIF-8/Ag_3PO_4 interface and the presence of an Ag NPs at the interfacial region. (B) Magnified HRTEM image of the selected Ag nanoparticle, revealing well-resolved lattice fringes. (C) Intensity profile obtained from the selected region in (B), showing an interplanar spacing of 0.238 nm, consistent with the (111) planes of face-centered cubic metallic Ag (Ag^0).
